# Supplementary material for: XMEA: A New Hybrid Diamond Multielectrode Array for the In Situ Assessment of the Radiation Dose Enhancement by Nanoparticles
Source: Sensors (Basel). 2024 Apr 10;24(8):2409. doi: 10.3390/s24082409 (PMC11053603; doi:10.3390/s24082409)
Supplement: Supplementary file 1 [file sensors-24-02409-s001.zip › sensors-2930022-supplementary.pdf]

Supplementary Material of the Article:

XMEA: A New Hybrid Diamond Multielectrode Array for the In Situ Assessment of the Radiation Dose Enhancement by Nanoparticles

Patricia Nicolucci [1], Guilherme Gambaro [1], Kyssylla Monnyelle Araujo Silva [1], Iara Souza Lima [1], Oswaldo Baffa [1] and Alberto Pasquarelli [2,*]

Electrode characterization.

The XMEA-electrodes were first tested by means of cyclic voltammetry (CV) in pure phosphate buffered saline (PBS) in order to test their function, the width of the potential window and the background activity.

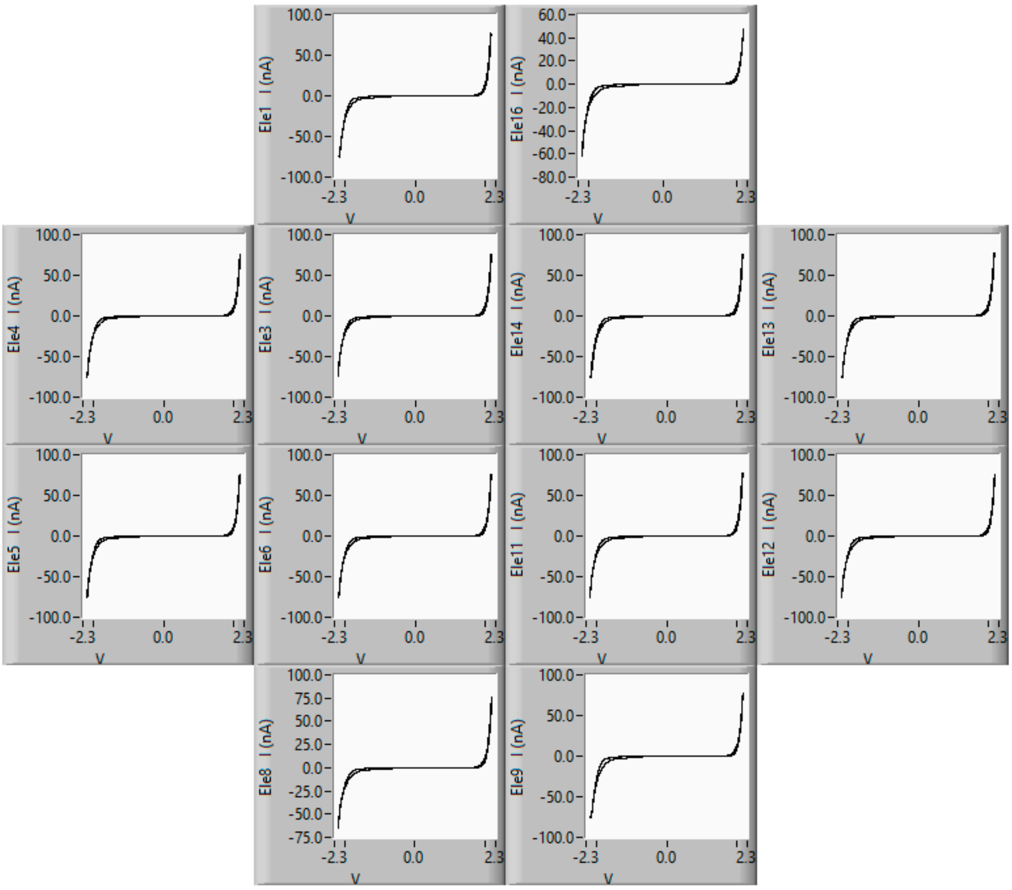

Figure S1. CV scan in PBS of the 12 active MEA-Channels, recorded at 50 mV/s. The water-splitting potential window is larger than 3 Volts. No significant background activity is observed.

The second test consisted in recording CV-plots with adrenaline diluted in PBS at different concentrations for assessing the sensitivity against this catecholamine. A software-controlled perfusion system was used for delivering adrenaline solutions of 3, 10, 30, 100, 300 and 1000 µM. First, the perfusion chamber of the device was filled with PBS, then the adrenaline solutions, ordered by increasing concentrations, were delivered in steps of 10 s in the perfusion chamber at a flow-rate of 0.2 mL/s, then the flow was stopped and the CV-response was recorded, at a scan rate of 50 mV/s. Out of these data it was possible to obtain a calibration function by taking the current values measured around 0.8 Volt of the rising ramp. The fit shows a good linearity with a slope of ~7 pA/µM. Figure S2 shows the results for one sample channel and the derived calibration plot with the linear fit.

The last characterization test, depicted in Figure S3, consists in noise measurements, which are important for assessing the limit of sensitivity by the recording of secretory events. This was performed as chronoamperometric measurements with a sampling rate of 4000 Hz, a bandwidth of 1 kHz obtained by means of an

analog Bessel-type low-pass filter of the 4th order. The recorded traces show a peak-to-peak noise of ~ 1 pA for all channels with the exception of channel 3. The lower panel represents the spectral noise density in the given bandwidth obtained by fast Fourier transform (FFT).

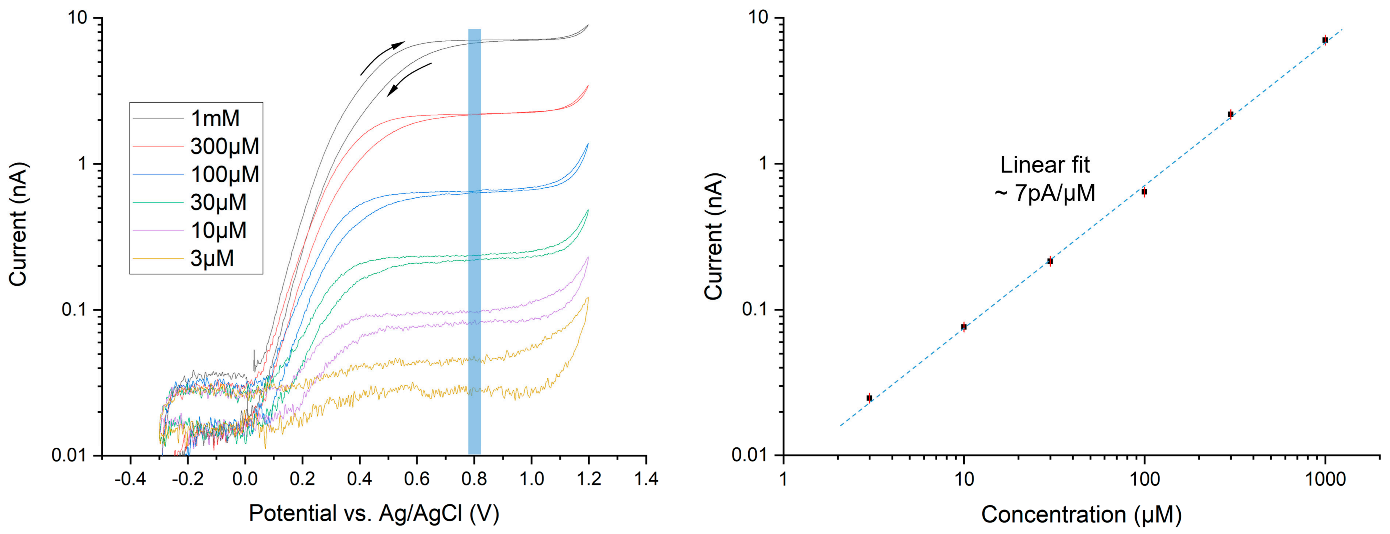

Figure S2. (Left) CV-scan with 6 different concentrations of adrenaline, recorded at 50 mV/s. The CV-curves are offset by 20 pA for avoiding negative values, which cannot represented in the logarithmic scale. The second slopes at the right end of the plots indicate the initial onset of the oxygen evolution due to the water splitting. (Right) Calibration plot derived from the CV-scan at left at 0.8 V of the rising ramp (shaded area), after subtracting the 20 pA offset. The fit shows a linear sensitivity of ~7 pA/µM.

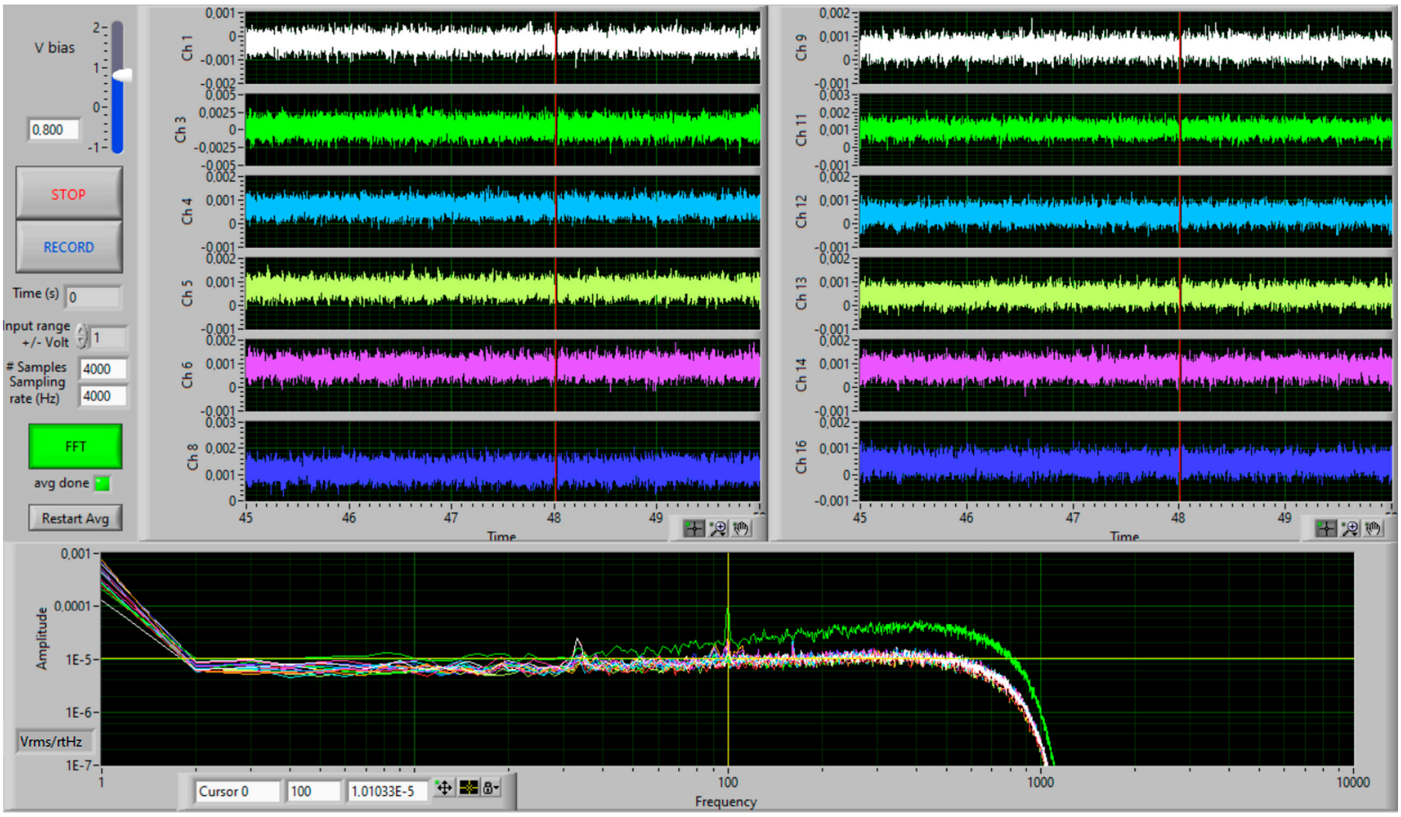

Figure S3. Noise test from the 12 active MEA-Channels biased at 800 mV. Channel 3 has a total noise of ~4 pA peak-to-peak (Y-scale in volts, 1 mV = 1pA). All other channels are at ~1pA total noise, including 50 and 100 Hz noise components (visible at the bottom in the FFT spectrum). DC background currents are visible as offsets and range from -0.5 to 1.5 pA. These noise levels allow the recording of exocytotic events from secretory cells with a limit of detection (LOD) of just a few pA.
